# The Identification of β-Ocimene Biosynthetic Pathway Through Mevalonate Acid (MVA) and 1-Deoxy-D-Xylulose 5-Phosphate (DXP) Pathways Using Crude Enzyme Extracts in Indonesian Bay Leaf/Salam Leaf (*Syzygium polyanthum*)

**DOI:** 10.21315/tlsr2022.33.2.1

**Published:** 2022-07-15

**Authors:** Bima Putra Pratama, Yudi Pranoto, Supriyadi, Respati Tri Swasono

**Affiliations:** 1 Department of Food and Agricultural Product Technology, Faculty of Agricultural Technology, Universitas Gadjah Mada, Jl. Flora, Bulaksumur, Yogyakarta 55281, Indonesia; 2 Department of Chemistry, Faculty of Mathematics and Natural Sciences, Universitas Gadjah Mada, Jl. Sekip Utara, Yogyakarta 55281, Indonesia

**Keywords:** Salam Leaf, β-ocimene, Mevalonic Acid Pathway, 1-deoxy-D-xylulose 5-phosphate Pathway

## Abstract

Salam leaf has a β-ocimene as a key volatile compound that gives a fresh aroma to the food when the salam leaves are involved in the cooking process. As a secondary metabolic product, enzymatic biosynthesis as the early stage of β-ocimene is a factor that needs to be known. Thus, this study was done to identify the mechanism of the two well-known terpenoid biosynthetic pathways, namely Mevalonate Acid (MVA) and 1-Deoxy-D-Xylulose 5-Phosphate (DXP) pathways, in the biosynthesis of β-ocimene in salam leaves. The activity of the 3-hydroxy-3-methylglutaryl coenzyme A reductase (HMGR)-MVA pathway-determining enzyme and 1-deoxy-D-xylulose-5-phosphate synthase (DXS)-DXP pathway-determining enzyme in the crude enzyme and their derivative products of salam leaves were analysed for their changes by differences of substrate ratios and enzyme inhibitors. The results showed that the activity of the HMGR enzyme was lower significantly than the DXS enzyme based on the addition of variations to the substrate ratio. These results were also supported by the enzyme and substrate reaction products, MVA and Isopentenyl diphosphate (IPP) intermediates from the MVA pathway, which were significantly lower when compared to DXP and IPP intermediates from the DXP pathway. As the end product of the reaction, β-ocimene gave a significantly higher value of the DXP pathway than the MVA pathway. Therefore, it can conclude that the mechanism of the biosynthetic pathway of β-ocimene in salam leaves was synthesised via the DXP pathway. The production of β-ocimene could have crosstalk-pathway through the MVA pathway, especially when the DXP pathway was blocked.

**Highlights**

- The activity of the enzyme that plays a role in the biosynthesis of terpenoid compounds, namely the DXS enzyme, has a higher activity than the HMGR enzyme in this research. Similarly, the total value of the resulting metabolite products, namely DXP, MVA and IPP. These results indicate the DXP pathways dominance in terpenoid biosynthesis in Salam leaves.
- The same graphic pattern on the total value of β-ocimene with the analysis of enzyme activity and other metabolite products due to variations in substrate concentrations explains that the biosynthesis of β-ocimene in this study occurs through the DXP pathway.
- The total value of β-ocimene that appeared in samples added with fosmidomycin inhibitor explains the crosstalk pathway mechanism in β-ocimene biosynthesis in Salam leaves.

## INTRODUCTION

Salam leaves have been used for a long time as an aroma enhancer for food in Indonesia (Yuwono & Faustina 2019). The aroma of salam leaves comes from the essential oil found in the leaves. It consists of 342 μg/mg of the oil of monoterpene hydrocarbons, 140 μg/mg of the oil of oxidised monoterpene, 260 μg/mg of the oil of sesquiterpene hydrocarbons, 215 μg/mg of the oil of oxygenated sesquiterpene and 43 μg/mg of the oil of others (Cock & Cheesman 2018). It is known that α-pinene and β-ocimene as the two main terpenoid compounds that give a floral and herbal aroma in the salam leaves (Eganathan *et al*. 2012). Of the two dominant compounds, the odor detection threshold of β-ocimene is 0.0034 μg/mg of the oil smaller than that of α-pinene at 0.19 μg/mg of the oil. Thus, β-ocimene is described as a key odorant in salam leaves. The concentration of 80 μg/mg– 100 μg/mg of the oil for β-ocimene is too small and fluctuates due to various factors. This impacts the character of the aroma produced (Suvarchala Reddy *et al*. 2015).

One of the factors determining the amount of β-ocimene in plant essential oil is the enzymatic biosynthesis process in the plant (Öztekin & Martinov 2013). There are two well-known pathways for the biosynthesis of terpenoid compounds. The first pathway is the mevalonic acid (MVA) pathway, which occurs in the cytosol, and the second pathway is the 1-deoxy-D-xylulose 5-phosphate (DXP) pathway, which occurs in the plastid (Tritsch *et al*. 2010). Each of these pathways has a rate-limiting enzyme, for example, the MVA pathway has HMG CoA reductase (HMGR), and the DXP pathway has DXP synthase (DXS) as its rate-limiting enzyme. In previous studies, the enzyme activity of HMGR and DXS in the plant was used to track the pathway followed in the biosynthesis process of a terpenoid compound. Related to that, some substrates were used, namely 3-hydroxy-3-methylglutaryl coenzyme A (HMG CoA) for the MVA pathway and pyruvic acid combined with D-glyceraldehyde 3-phosphate (GAP) for the DXP pathway (Hirata *et al*. 2016).

Besides, the metabolic products such as the initial enzyme products (MVA and DXP) was determined, and then the intermediate compound, which was the isopentenyl diphosphate (IPP) was then determined as well as to see the continuity of the reaction process up to the middle phase, and to see the fluctuation with initial enzyme activity, initial reaction products, and with target compounds as end products. Enzyme inhibitors (the pravastatin enzyme inhibitor was used for the MVA pathway and fosmidomycin was used for the DXP pathway) were usually involved to prove the existence of a cross-talk pathway (another pathway contribution when the main pathway is restricted) (Skorupinska-Tudek *et al*. 2008)In the final step, the quantification process on the target compound (the terpenoid compound) was carried out to match the fluctuations in the enzyme activity with the upstream product, intermediate, and downstream products (Keilwagen *et al*. 2017).

The tracking becomes important to do for developing purposes on a target compound widely by knowing its changes since the initial phase in plants. But, until now, the information is far from sufficient. Moreover, besides β-ocimene is an aroma compound and an anti-bacterial compound, β-ocimene is also a phytohormone compound that plays a role in plant growth and maturity (Kang *et al*. 2018). Therefore, this research conducted to identify the β-ocimene biosynthetic pathway in salam leaves by the variance of substrate ratios of the pathway and enzyme inhibitors on the changes of enzyme activity and the metabolic products (initial product, intermediate product and end product) of the pathway resulted.

## MATERIALS AND METHODS

### Materials

All steps in this research were completed at the Faculty of Agricultural Technology, Universitas Gadjah Mada, Yogyakarta, Indonesia. The main material was salam leaves obtained from CV Bina Agro Mandiri, Yogyakarta, Indonesia (–7.827300, 110.350024). Leaves were selected from the eighth leaf of the shoot without any visual damage from pests. The leaves used were the leaves with the same length and width for 9 cm × 5 cm. Leaves were washed and air-dried for 5 min at room temperature. Based on the thermogravimetric method, the leaves used in this study had an average moisture content of 70.15 ± 0.10% wet basis. The leaves were stored below 4°C if they were not yet to be used. All of the chemicals used in this research were purchased from Merck KGaA (Darmstadt, Germany).

### Treatments

There were two treatments as independent variables in this study, first was the substrate ratio of HMG CoA as a substrate-specific of the MVA pathway and the combination between pyruvate and GAP as a substrate-specific of the DXP pathway. The ratios of HMG CoA: pyruvate: GAP were 0: 0: 0 (A); 0: 8: 8 (B); 10: 6: 6 (C); 20: 4: 4 (D); 30: 2: 2 (E); or 40: 0: 0 (F) mg/g of the salam leaves crude enzyme extract. The second was sample variations, which were sample (salam leaves crude enzyme extract) plus substrate (SR), sample plus substrate plus pravastatin-HMGR inhibitor (SRIP), and sample plus substrate plus fosmidomycin-DXS inhibitor (SRIF).

### Crude Enzyme Preparation

The crude enzyme was extracted from the leaves by adding 25 g of salam leaves to 100 mL of cold acetone at 4°C. The mixture was blended by Philips Blender 5000 Series HR2222 four times with a minute pause every 30 sec of blending. The result was left for 5 min at room temperature before being centrifuged with an LMC-3000 Laboratory Centrifuge at 1000 g for 30 min. After the centrifugation process was done, the supernatant was dried with 25 g of calcium chloride overnight. Then, the dry sample was mixed into 50 mL of solution with aquades, 0.35 g of potassium dihydrogen phosphate, and 0.02 M glutathione powder and incubated for 1 h above ice water. After that, the mixture was centrifuged for 30 min. Its supernatant was taken as the salam leaves crude enzyme extract with a 40 mL volume and 20 mg of the dissolved protein/g of the crude enzyme extract (Hartanti *et al*. 2019).

### Metabolite Product Extraction

Crude enzyme extract was mixed with the buffer and some reagent kits similar to those used when measuring HMGR and DXS enzymes. In each of the SRIP and SRIF sample groups, the mixture was mixed with pravastatin and fosmidomycin, respectively, as much as 0.5 mg/g of crude enzyme. The mixture was incubated for 24 h with dark and light phases. After that, it was continued with the extraction process of the reaction product with 30 mL of cold acetone and macerated for 1 h. The mixture was again centrifuged at 3000 g for 30 min. The supernatant was a salam leaf metabolite product resulted from the crude enzyme and the substrate and was used as an analyte which was mentioned as metabolite product extract (MPE) to determine the total amount of the initial, intermediate, and final metabolite products. Meanwhile, the activities of HMGR and DXS be checked beforehand after the crude enzyme extraction process is completed (Hidayat *et al*. 2018).

### HMGR Enzyme Activity Assay

HMGR enzyme activity of the salam leaves crude enzyme extract (SLCEE) was determined by an enzyme kit consisting of buffer solution, NADPH coenzyme, HMG CoA substrate, HMGR enzyme, and enzyme inhibitor, namely, pravastatin. One milliliter of the SLCEE was mixed with 920 μL of the buffer solution diluted with ultrapure water at a ratio of 1:4, 20 μL of NADPH which was already dissolved in 1.5 mL of premade buffer, and 60 μL of HMG CoA. Then, after 5 min of incubation, enzyme activity was measured with a Thermo Scientific™ GENESYS 10S UV-Vis spectrophotometer at 37°C with λ = 340 nm. Enzyme activity was calculated using the following formula:


$$\text{Enzyme activity }(\text{U}/\text{mL Protein})=\frac{\begin{array}{l} (\text{A}340\;\text{sample of SLCEE}-\text{A}340\;\text{blank}\\ \text{sample without HMGR})\times \text{Volsample} \end{array}}{\left(12.44\times 0.6\times \text{LP}\right)}$$

Where, 12.44 = the extinction coefficient of NADPH; 0.6 = the concentration enzyme in the protein; LP = the light path; and cuvette= 1 (Hartanti *et al*. 2019).

### DXS Enzyme Activity Assay

DXS enzyme activity of the SLCEE was determined by mixing one milliliter of SLCEE with 100 μL of HEPES buffer, 1 mL of bovine serum albumin solution, 5 μL of magnesium chloride solution, 2.5 μL of TCEP (tris(2-carboxyethyl)phosphine), 1 μL of thiamine diphosphate, 100 μL of coenzyme NADPH, 1 μL of IspC coupled enzyme solution, and 1 mL of GAP and pyruvic acid solution each to reach the final volume of 3 mL solution. Then, the mixture was left at 37°C for 5 min. The rate of NADPH reduction produced a change in absorbance that measured by spectrophotometry every 30 sec for 5 min at λ = 340 nm at 37°C to determine the specific activity of the DXS enzyme. The activity of DXS was calculated using the following formula:


$$\text{Enzyme activity }(\text{U}/\text{mL Protein})=\frac{\begin{array}{l} (\text{A}340\;\text{sample of SLCEE}-\text{A}340\;\text{blank}\\ \text{sample without HMGR})\times \text{Volsample)} \end{array}}{\left(12.44\times 1.05\times \text{LP}\right)}$$

Where, 12.44 = the extinction coefficient of NADPH; 1.05 = the concentration enzyme in the protein; LP = the light path; cuvette = 1 (Brammer *et al*. 2011).

### The MVA and DXP Products Quantification

The MVA quantification was done by added one milliliter of MPE to 2.5 mL of methanol, 1 mL of 0.1 N HCl, 0.5 mL of distilled water, and then vortexed. The mixture was incubated for 30 min to convert MVA to MVAL. Then, it was transferred to a Thermo Scientific™ HyperSep™ C18 Cartridges cartridge (conditioned with 1 mL of methanol and 1 mL of 0.1 N HCl). The cartridge was dried for 3 min, and the analyte was then eluted with 3x1 mL of 15% methanol in water. The residue was dissolved in 10 mL of 0.2% ammonium hydroxide to convert MVAL into MVA. The solution resulted was material for analysis using LC-MS. Quantification was performed using standard MVA curves from five concentration series in the range 20 mg/g–100 mg/g of MVA standard solution (Zhou *et al*. 2012).

For the DXP quantification, a 1 mL MPE was extracted with 5 mL of 10% cold methanol. Then, the mixture was vortexed for 3 min and centrifuged at 3000 g for 30 min at 4°C. The supernatant was taken and filtered with a 3,000-MW-cutoff (MWCO) Amicon Ultra 0.5 mL filter. The filtrate was used as the analyte of the quantification of the DXP by Waters® ACQUITY™ TQD liquid chromatography-mass spectrometry (LC-MS).

### Isopentenyl Diphosphate (IPP) Product Assay

Total IPP was determined by using a UV-Vis spectrophotometer. The steps were a total of 10 μL of the MPE, 2.5 μL of farnesyl pyrophosphate (FPP), 5 μL geranylgeranyl pyrophosphate (GGPP), 610 μL of FPP synthase, 80 μL of GGPP synthase, and 1 mL of 50% Tris-HCl was mixed and incubated for 120 min at 30°C. Then, the reaction was stopped with the addition of 500 μL of acetonitrile and 50 μL of 10% hydrogen chloride. The mixture then was left for 24 h at 4°C. The denatured protein was removed by centrifugation at 3,000 g at 4°C for 30 min, and the supernatant was measured at a wavelength of 370 nm. The formula used to determine the total IPP is as follows:


$$\text{Total IPP }(\text{mg}/\text{g of the MPE})=\frac{\begin{array}{l} (\text{Abs}370\text{MPA sample}-\text{Abs}370\;\text{blank}\\ \text{without MPA sample})\times \text{(17}\text{.43}+\text{4}\text{.63)} \end{array}}{\left(1.49\times \text{Vol sample in mL}\right)}$$

Where, 17.9 = FPP extinction coefficient; 4.63 = GGPP extinction coefficient; 0.49 = IPP extinction coefficient; Vol = Volume sample (Rothman *et al*. 2007).

### β-ocimene Quantification

As a metabolic end product, the β-ocimene was obtained from the essential oil profiles of 5 mL of MPE which was water-distillated by using 20 mL of water for 1 h. One microliter of the essential oil was used for analysis and to be injected into gas chromatography-mass spectrometry (GC-MS). The β-ocimene quantification was carried out based on the ocimene standard in hexane at five series concentrations of 50 mg/g–250 mg/g. The specifications for the analysis were Shimadzu GC17A MS QP-5000 GC-MS. The GC-MS was equipped with a CP Sil 5 CB column, ionising electron impact, ionisation energy of 70 eV, with a 60°C initial column temperature and a 250°C final temperature, a 10°C/min of the temperature rate, and helium as the carrier gas (Vrhovsek *et al*. 2014).

### Statistical Analysis

This research was conducted with a completely randomised design. The data obtained were analysed statistically by using variance analysis (ANOVA), and the data that significantly different was then analysed by Duncan Multiple Range Test (DMRT) with 95% significance level.

## RESULTS AND DISCUSSIONS

### HMGR and DXS Enzyme Activities

**Figure 1:**
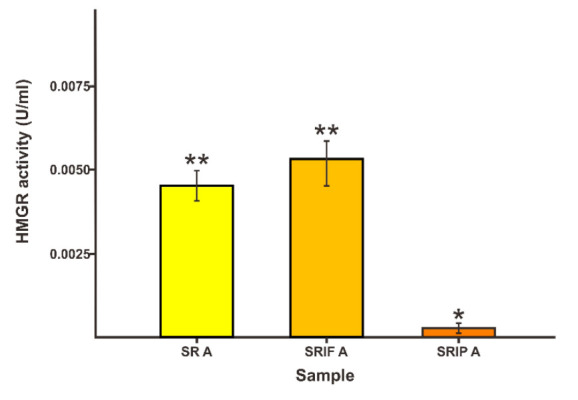

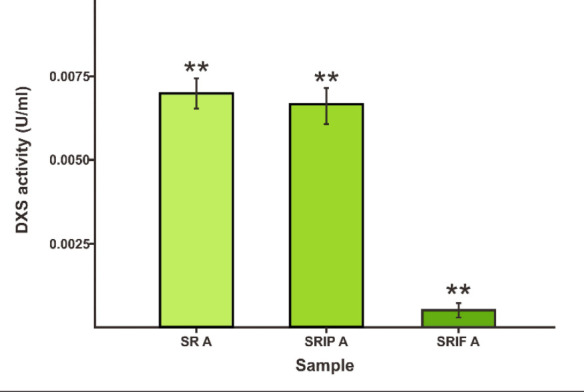
HMGR (a) and DXS (b) enzyme activities (U/mL protein) in sample A with substrate ratio of HMG CoA/[Pyruvate/GAP] = 0/[0/0] or sample without substrate. The number of asterisks (*) indicates a significant difference in the activity of each sample (*p*<0.05).

In Fig. 1, the HMGR and DXS enzyme activities of salam leave crude enzyme extracts (SLCEE) are presented. The HMGR and DXS enzyme activities of the SR, SRIF, SRIP in A samples were analysed to observe the activity of the enzymes in SLCEE without any substrate induced. Sample A data was used to see the normality of the enzyme activity of HMGR and DXS in salam leaves. The results were 0.0050 U/mL–0.0115 U/mL protein (for HMGR enzyme activity) and 0.0075 U/mL –0.0125 U/mL protein (for DXS enzyme activity). Based on the literature, the enzyme activity of HMGR and DXS were in the ranges of 0.0100 U/mL to 0.0300 U/mL and 0.0075 U/mL to 0.0120 U/mL protein, respectively (Sharma *et al*. 2015; Wright & Phillips 2014). The data was slightly below the literature. This may be due to the different types of organisms affect to their enzyme activities (Wei *et al*. 2019).

Besides, it can be seen that SR-A and SRIF-A samples were not significantly different (0.0058a U/mL and 0.0052a U/mL protein for the HMGR enzyme), and SR-A and SRIP-A were also not significantly different (0.0070a U/mL and 0.0066b U/mL protein for the DXS enzyme). According to theory, the targeted enzyme drops dramatically due to enzyme inhibitors, both of which are competitive inhibitors. (Sharma *et al*. 2015). The data mentioned was used to see the enzyme activity for its normality in nature. The use of an inhibitor in the sample was used to ensure that the undue inhibitor has no effect on the activity of the enzyme involved.

**Figure 2:**
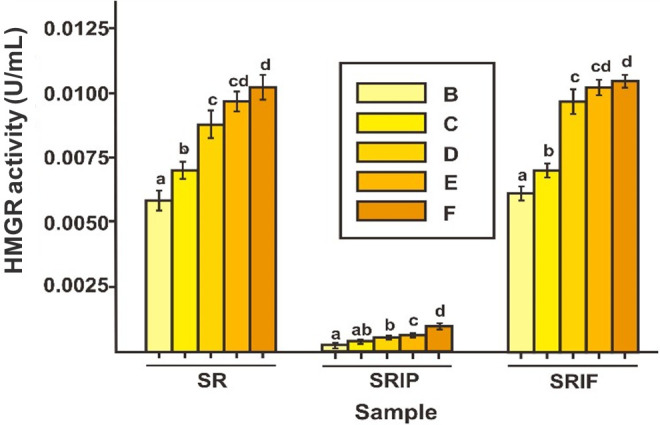

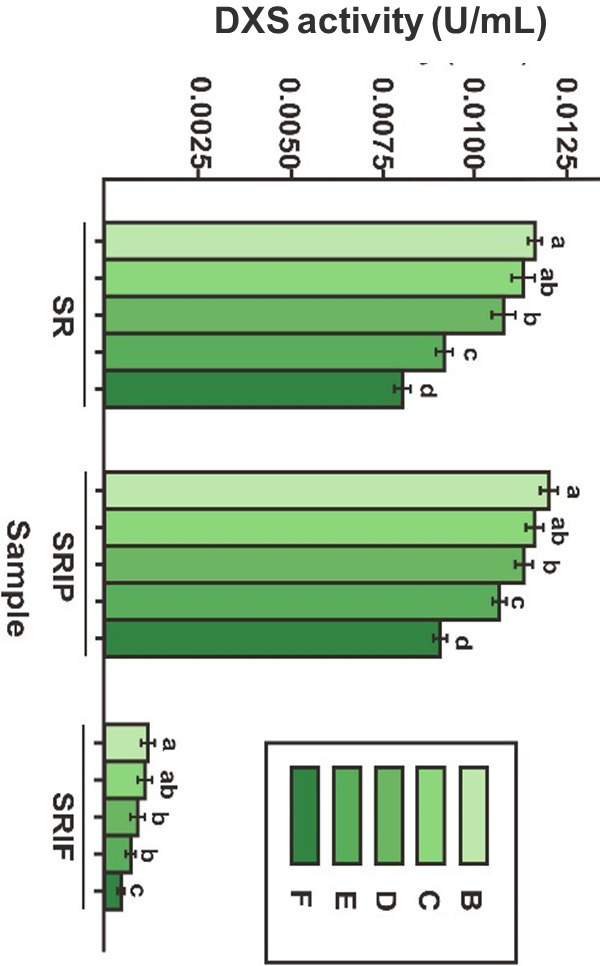
HMGR (a) and DXS (b) enzymes activity (U/mL protein) with substrate ratio contribution of HMG CoA/[Pyruvate/GAP] = 0/[8/8] (sample-B); 10/[6/6] (sample-C); 20/[4/4] (sample-D); 30/[2/2] (sample-E); and 40/[0/0] (sample-F) mg/g of the SLCEE. Lowercase indicates a significant difference in the activity of each sample in one group sample (*p*<0.05).

Afterward, Fig. 2 showed the difference in the changes of HMGR and DXS enzyme activity when the substrate was involved. The sample in Fig. 2 were SR, SRIP, and SRIF for –B, –C, –D, –E and –F samples with the use of variations in the substrate ratio described in the method. The results showed that both the HMGR enzyme and the DXS enzyme activities were increased significantly by the increasing of a substrate that was added. HMGR enzyme activity increased with the addition of β-Hydroxy β-methylglutaryl-CoA (HMG-CoA) substrate, while DXS enzyme activity increased with the increase of the amount of pyruvate and GAP substrates. SRIF and SRIP sample data can be seen in Fig. 2, for the HMGR enzyme, the activity has decreased drastically in the SRIP sample, while the SRIF sample did not change when compared to the SR sample. This also happened to the DXS enzyme, its activity decreased significantly in the SRIF sample, while there was no change in the SRIP sample. The specific substrates and enzyme inhibitors involved in this study do not interfere with other pathways (Skorupinska-Tudek *et al*. 2008).

The highest activity of the HMGR enzyme was in the SRIF-F sample with the enzyme activity of 0.0114 U/mL protein. This result was obtained by the addition of the HMG-CoA substrate of the MVA pathway. It was lower significantly with the enzyme activity value of the DXS enzyme in the DXP pathway with the addition of pyruvate and GAP substrates with the maximum value of enzyme activity in the SRIP-B sample of 0.0123 U/mL protein. Thus, from the data obtained, it is known that in SLCEE the activity of the DXS enzyme is higher than that in the HMGR enzyme. This was explained, with higher levels of monoterpenoid than sesquiterpenoid in leaves of the plant, it was possible that the DXS enzyme activity was higher than the HMGR enzyme as an enzyme that should be the main producer of monoterpenoid (Tritsch *et al*. 2010).

### Total MVA and DXP (Initial Product of Metabolic Pathway)

**Figure 3:**
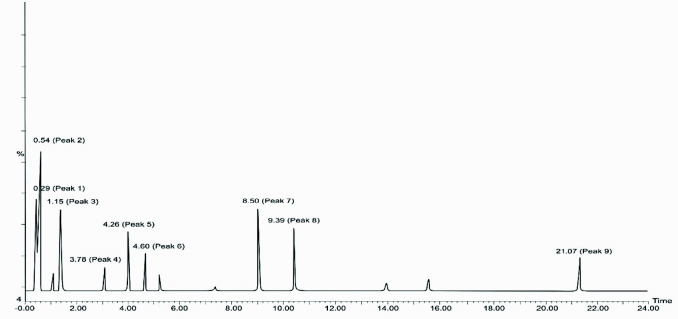
Chromatogram of non-volatile compounds of salam leaf extract.

**Figure 4:**
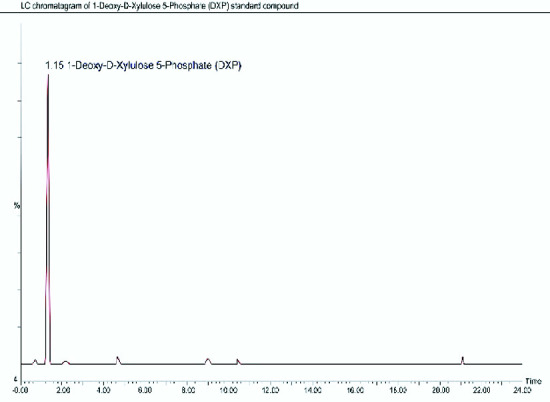

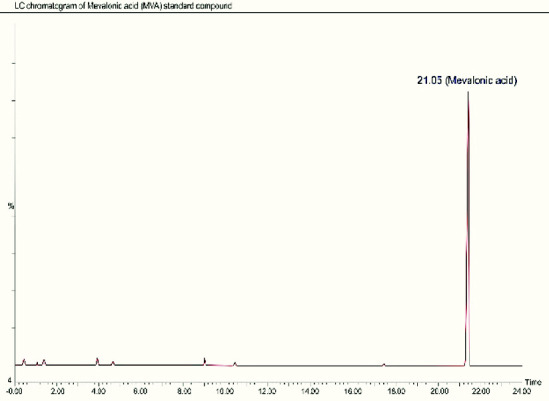
LC-chromatograms of the DXP (a) and MVA (b) standards which are at the same peak, in the third and ninth peak of the salam leaf extract chromatogram for quantification of MVA and DXP of the MPE.

The identification of the initial MVA and DXP products was run by using LC-MS. In the LC chromatogram resulted (Fig. 3), there were nine peaks presented in the profile of non-volatile compounds from the salam leaves extract without any treatments. The peaks were matched with the database from the NIST Standard Reference Database number 69 (2018) and produced descriptions of non-volatile compounds in Table 1. To quantify the total MVA and DXP, the MVA and DXP standards were also analysed by LC-MS which produced a chromatogram in Fig. 4. The results on the MVA and DXP standards were consistent with the literature, DXP is a compound that is less polar than MVA (Miziorko 2011). This research was run in reverse phase LC-MS.

**Figure 5:**
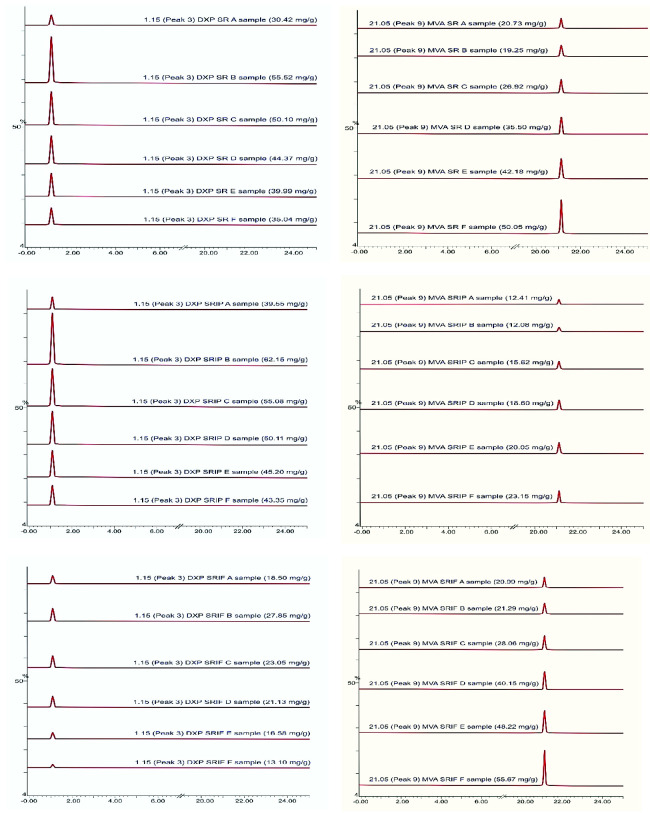
Chromatograms of the DXP (a, b, c) and MVA (d, e, f) compounds in mg/g salam leaves MPE extract of SR, SRIP, and SRIF –A (samples without the use of substrates) and –B to –F samples (samples with different substrate ratios).

After the standard analysis process of MVA and DXP has been completed, the process is continued with LC-MS analysis of non-volatile compounds from SR, SRIF, and SRIP –A, –B, –C, –D, –E and –F samples. The results can be seen in Fig. 5. Based on the chromatogram in Fig. 5, DXP compounds are found at the third peak with a retention time (Rt) of 1.15 min and m/z 225. Then, for the MVA compound, it is at the ninth peak with a Rt of 21.05 min and m/z 132. Fig. 5 shows an increase in the MVA product along with the addition of the HMG-CoA substrate. This happened in all samples of SR, SRIF, and SRIP with the highest values, namely 50.05, 55.67, 23.15 mg/g of the MPE. This also happened to the total DXP in SR, SRIP, and SRIF samples with the highest values achieved at 55.52, 62.15, and 27.85 mg/g of the MPE. These results indicate that the initial metabolite product, namely DXP, is higher than MVA. These results are consistent with the results of DXS and HMGR enzyme activity in this study (Hampel *et al*. 2005). The mechanism for the formation of MVA compounds in the mevalonic acid pathway is that the MVA pathway in plants will take place and occur in the cytosol. Then, the process is first in the presence of the primary substrate and the first, namely, two acetyl-CoA, which is then combined with the help of the enzyme AACT (acetoacetyl-CoA-thiolase). After that, the process was continued by adding one more molecule of acetyl-CoA to form HMG-CoA with the synthase enzyme, namely HMGS (HMG-CoA-synthase). This mechanism was then continued by the enzyme tested in this study, namely the HMGR enzyme, which reduces HMG-CoA compounds to MVA. This HMGR enzyme is the critical enzyme in the MVA pathway as the leading substrate supplier, namely MVA. So, to trace the mechanism of terpenoid compounds in the MVA pathway, HMGR activity needs to be known (Rehman *et al*. 2016).

Fig. 5 also showed that the peak produced between samples in the group of DXP results, and the peak is slightly higher for SRIP when compared to the SR sample. For example, SRIP B had a higher peak than SR B (62.15 mg/g with 55.52 mg/g of the MPE). However, for SRIF B, it was 27.85 mg/g of MPE, which was very low due to fosmidomycin, which was effective against DXS. The difference in peaks produced in Fig. 5 is similar. The SRIP F sample (with pravastatin) was the sample with the lowest peak (23.15 mg/g of MPE) when compared to the SR and SRIF F samples (50.05 mg/g with 55.67 mg/g of MPE). Samples B and F in the DXP and MVA sample groups were compared because of their maximum equivalents for their specific substrate ratios. Meanwhile, in the DXP pathway, the mechanism for forming DXP compounds is the reaction begins with the condensation of pyruvic acid with glyceraldehyde-3-phosphate to form DXP, which is catalysed by the DXS enzyme. DXP compounds are compounds that play an essential role in the DXP pathway as the primary substrate. So its presence needs to be analysed to see the course of the DXP pathway in the metabolic process of terpenoid compounds (Baidoo *et al*. 2014).

### Total IPP (Intermediate Product Metabolic Pathway)

The IPP intermediate compound in the biosynthetic process needs to be examined to ensure the continuity of a biosynthetic mechanism, to see which biosynthetic pathway is more dominant, and to provide a pattern to be matched with the downstream product pattern. The total IPP is shown in Fig. 7.

**Figure 6:**
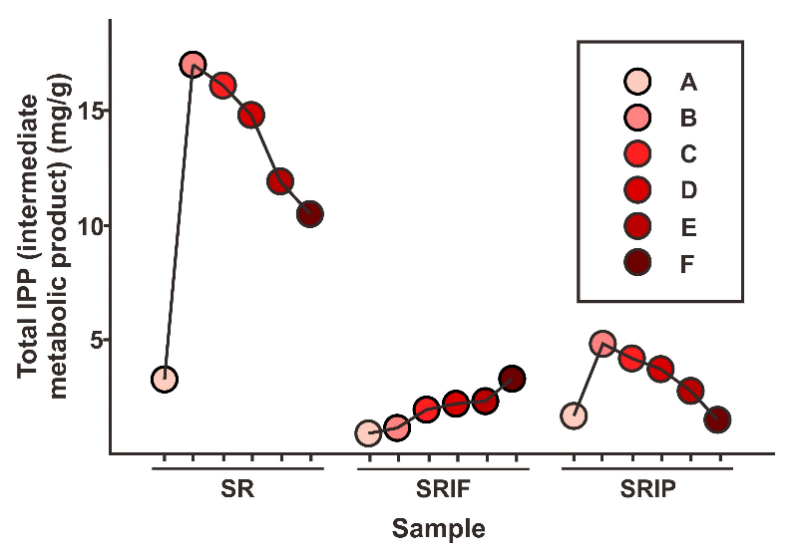
Graph of total IPP (intermediate compound) in the mg/g salam leaves MPE extract of the SR, SRIP, and SRIF –A (samples without the use of substrates) and –B to –F samples (samples with different substrate ratios).

The results in Fig. 6 indicate the continuation of the biosynthetic reaction carried out in this research. There was an increase in the total IPP along with the increase in the amount of substrate added to the system with the crude enzyme extract of salam leaf. However, the increase tends to follow the pattern of the addition of the DXP line substrate. In the SR sample, the total IPP was at a value of 11.05 mg/g to 18.35 mg/g MPE. This value increases from samples F, E, D, C to sample B.

Then, with the use of an enzyme inhibitor, fosmidomycin, the samples were in the concentration range of 1.16 mg/g to 5.15 mg/g of the MPE. Whereas in the use of pravastatin inhibitors, the total IPP value was in the range of 0.30 mg/g to 2.50 mg/g of the MPE. Thus, it can be said that the activity of the DXP pathway is higher, as seen from the use of pravastatin inhibitors, which provide a higher level of IPP product than fosmidomycin inhibitors. For IPP intermediate product, the metabolism is different between the MVA and DXP pathways. The MVA pathway goes through phosphorylation and decarboxylation and becomes IPP, while in the DXP pathway, the process goes through continuous phosphorylation and reduction to become IPP. But the two pathways converge to form IPP compounds, and the reason is that IPP is the backbone of terpenoid compounds. So that the analysis of these compounds needs to be carried out to determine the sustainability of the metabolic scheme of the two pathways to the middle part of the metabolic process of terpenoid compounds (Kuzuyama & Seto 2012). These results are explained in the research of Wright and Phillips (2014). The activity of this enzyme can only be measured using highly sensitive analytical equipment. Here, a method is described to determine the DXS enzyme activity in a crude plant extract, by measuring DXP production directly using high performance liquid chromatography linked to a tandem triple quadrupole mass spectrometry detector (LC-MS/MS, in which plants have the possibility of a dominant DXP pathway if their monoterpenoid composition is higher than the number of groups of sesquiterpenoid compounds. The total IPP obtained in the data on the use of pravastatin proves the existence of a crosstalk pathway in this research.

### Total β-Ocimene (End Product of Metabolic Pathway)

**Figure 7:**
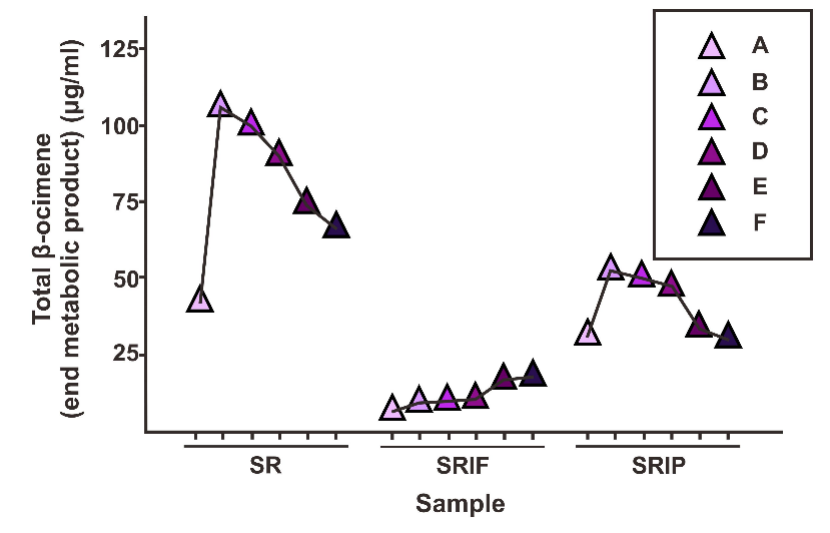
Graph of the total β-ocimene (intermediate compound) in μg/mL salam leaves MPE essential oil of SR, SRIP, and SRIF –A (samples without the use of substrates) and –B to –F samples (samples with different substrate ratios).

Fig. 7 shows the pattern of changes in the total end products of the β-ocimene. There was an increase in the total β-ocimene especially in SR samples which had a value range from 42.15 μg/mL to 102.10 μg/mL of MPE essential oil. The pattern of total β-ocimene increase was also linear with the addition of the DXP line substrate. The SRIF sample has a value range of 5.27 μg/mL to 17.45 μg/mL of MPE essential oil. Meanwhile, the SRIP sample has a value range of 28.17 μg/mL to 54.26 μg/mL of MPE essential oil. Thus, the use of a fosmidomycin inhibitor has more effect on the total β-ocimene produced. It has also been explained in previous studies, that plant essential oil monoterpenoid tend to be produced in the DXP pathway rather than the MVA pathway (Tritsch *et al*. 2010).

Fig. 7 also shows the possibility of a crosstalk pathway in the β-ocimene biosynthesis mechanism. This is indicated by the total value of b-ocimene in the SRIF sample which has increased along with the addition of HMG-CoA substrate. However, this value is significantly lower when compared to the values in the SR and SRIP samples. Thus, there is still a chance for a crosstalk pathway to synthesize β-ocimene in salam leaves. In closing, when the data in Fig. 7 is compared to the other data in this research, the biosynthetic process identified herein occurs through the DXP pathway. It was explained in the previous research design has also been proven that monoterpenoid compounds and their derivatives tend to be produced through the DXP pathway rather than the MVA pathway and this is done in *Arabidopsis thaliana* plant (Carretero-Paulet *et al*. 2006).

## CONCLUSION

Based on the data, the HMGR salam leaf crude enzyme extract and metabolite products from the MVA pathway have increased along with the HMG-CoA substrate ratio. Likewise, the activity of the DXS salam leaf crude enzyme extract and its metabolite products increased along with the increasing ratio of pyruvate and GAP substrates. It can be seen from the results that the activity of the DXS enzyme is higher than that of the HMGR enzyme. The initial DXP product also has a higher value than the value of the MVA product. It was followed by the value of IPP and β-ocimene intermediate products. In addition, the use of fosmidomycin causes a decrease in the total value of β-ocimene. Therefore, it concluded that β-ocimene is produced from the DXP pathway. Besides, the use of pravastatin indicates the possibility of a crosstalk pathway with β-ocimene being produced in small amounts. These results also can be used as a reference in the exploration for increasing the total value of β-ocimene enzymatically or genomically.

## Figures and Tables

**Table 1:** The description of non-volatile compounds in salam leaf extract. Rt = retention time; (m/z) = molecular ion, from the peaks of the chromatogram of salam leaf extract.

Peak	Rt (min)	(m/z)	Product fragment ion	Name
Peak 1	0.29	382	382, 365, 265, 219, 204, 175, 150, 111, 104, 94, 83, 60	Docosahexanoic acid
Peak 2	0.54	166	166, 120, 100, 83, 60	Valeric acid
Peak 3	1.15	225	225, 214, 206, 188, 169, 152, 139, 122, 114, 102, 99, 83, 61	Deoxyxylulose phosphate
Peak 4	3.78	319	319, 284, 197, 169, 125, 122, 111, 83, 60	Isonicotinic acid
Peak 5	4.26	360	360, 340, 299, 169, 127, 11, 83, 60	Malonic acid
Peak 6	4.60	398	398, 169, 139, 123, 97, 83, 60	Fumaric acid
Peak 7	8.50	319	319, 291, 276, 231, 123, 84, 61, 60	Oxalic acid
Peak 8	9.39	289	289, 203, 122, 83, 61, 60	Decanoic acid
Peak 9	21.07	132	132, 123, 84, 83, 60	Mevalonic acid

## References

[b1-tlsr-33-2-1] Baidoo EEK, Xiao Y, Dehesh K, Keasling JD (2014). Metabolite profiling of plastidial deoxyxylulose-5-phosphate pathway intermediates by liquid chromatography and mass spectrometry. Methods in molecular biology.

[b2-tlsr-33-2-1] Brammer LA, Smith JM, Wades H, Meyers CF (2011). 1-Deoxy-D-xylulose 5-phosphate synthase catalyzes a novel random sequential mechanism. Journal of Biological Chemistry.

[b3-tlsr-33-2-1] Carretero-Paulet L, Cairó A, Botella-Pavía P, Besumbes O, Campos N, Boronat A, Rodríguez-Concepción M (2006). Enhanced flux through the methylerythritol 4-phosphate pathway in Arabidopsis plants overexpressing deoxyxylulose 5-phosphate reductoisomerase. Plant Molecular Biology.

[b4-tlsr-33-2-1] Cock IE, Cheesman M, Goyal MR, Ayeleso AO (2018). Plants of the genus Syzygium (Myrtaceae): A review on ethnobotany, medicinal properties and phytochemistry. Bioactive Compounds of Medicinal Plants: Properties and Potential for Human Health.

[b5-tlsr-33-2-1] Eganathan P, Saranya J, Sujanapal P, Parida A (2012). Essential oil composition of leaves of Syzygium makul Gaertn. Journal of Essential Oil Bearing Plants.

[b6-tlsr-33-2-1] Hampel D, Mosandl A, Wüst M (2005). Biosynthesis of mono- and sesquiterpenes in carrot roots and leaves (*Daucus carota* L.): Metabolic cross talk of cytosolic mevalonate and plastidial methylerythritol phosphate pathways. Phytochemistry.

[b7-tlsr-33-2-1] Hartanti L, Yonas SMK, Mustamu JJ, Wijaya S, Setiawan HK, Soegianto L (2019). Influence of extraction methods of bay leaves (*Syzygium polyanthum*) on antioxidant and HMG-CoA Reductase inhibitory activity. Heliyon.

[b8-tlsr-33-2-1] Hidayat Y, Hermawati E, Setiasih S, Hudiyono S, Saepudin E (2018). Antibacterial activity test of the partially purified bromelain from pineapple core extract (*Ananas comosus* [L.] Merr) by fractionation using ammonium sulfate acetone. AIP Conference Proceedings.

[b9-tlsr-33-2-1] Hirata H, Ohnishi T, Watanabe N (2016). Biosynthesis of floral scent 2-phenylethanol in rose flowers. Bioscience, Biotechnology and Biochemistry.

[b10-tlsr-33-2-1] Kang ZW, Liu FH, Zhang ZF, Tian HG, Liu TX (2018). Volatile β-ocimene can regulate developmental performance of peach aphid myzus persicae through activation of defense responses in chinese cabbage brassica pekinensis. Frontiers in Plant Science.

[b11-tlsr-33-2-1] Keilwagen J, Lehnert H, Berner T, Budahn H, Nothnagel T, Ulrich D, Dunemann F (2017). The terpene synthase gene family of carrot (*Daucus carota* L.): Identification of QTLs and candidate genes associated with terpenoid volatile compounds. Frontiers in Plant Science.

[b12-tlsr-33-2-1] Kirby J, Nishimoto M, Chow RWN, Baidoo EEK, Wang G, Martin J, Schackwitz W, Chan R, Fortman JL, Keasling JD (2015). Enhancing Terpene yield from sugars via novel routes to 1-deoxy-D-xylulose 5-phosphate. Applied and Environmental Microbiology.

[b13-tlsr-33-2-1] Kuzuyama T, Seto H (2012). Two distinct pathways for essential metabolic precursors for isoprenoid biosynthesis. Proceedings of the Japan Academy, Series B.

[b14-tlsr-33-2-1] Miziorko HM (2011). Enzymes of the mevalonate pathway of isoprenoid biosynthesis. Archives of Biochemistry and Biophysics.

[b15-tlsr-33-2-1] Öztekin S, Martinov M (2013). Medicinal and aromatic crop drying. Stewart Postharvest Review.

[b16-tlsr-33-2-1] Rehman R, Hanif MA, Mushtaq Z, Al-Sadi AM (2016). Biosynthesis of essential oils in aromatic plants: A review. Food Reviews International.

[b17-tlsr-33-2-1] Rothman SC, Helm TR, Poulter CD (2007). Kinetic and spectroscopic characterization of type ii isopentenyl diphosphate isomerase from *Thermus thermophilus*: Evidence for formation of substrate-induced flavin species. Biochemistry.

[b18-tlsr-33-2-1] Sharma SN, Jha Z, Sinha RK, Geda AK (2015). Jasmonate-induced biosynthesis of andrographolide in *Andrographis paniculata*. Physiologica Plantarum.

[b19-tlsr-33-2-1] Skorupinska-Tudek K, Wojcik J, Swiezewska E (2008). Polyisoprenoid alcohols: Recent results of structural studies. The Chemical Record.

[b20-tlsr-33-2-1] Suvarchala Reddy NVL, Aveti S, Anjum M, Ganga Raju M (2015). Anti-hyperlipidemic activity of methanolic extract of syzygium alternifolium bark against high-fat diet and dexamethasone-induced hyperlipidemia in rats. Asian Journal of Pharmaceutical and Clinical Research.

[b21-tlsr-33-2-1] Tritsch D, Hemmerlin A, Bach TJ, Rohmer M (2010). Plant isoprenoid biosynthesis via the MEP pathway: In vivo IPP/DMAPP ratio produced by (E)-4-hydroxy-3-methylbut-2-enyl diphosphate reductase in tobacco BY-2 cell cultures. FEBS Letters.

[b22-tlsr-33-2-1] Vrhovsek U, Lotti C, Masuero D, Carlin S, Weingart G, Mattivi F (2014). Quantitative metabolic profiling of grape, apple and raspberry volatile compounds (VOCs) using a GC/MS/MS method. Journal of Chromatography B: Analytical Technologies in the Biomedical and Life Sciences.

[b23-tlsr-33-2-1] Wei H, Xu C, Movahedi A, Sun W, Li D, Zhuge Q (2019). Characterization and function of 3-Hydroxy-3-Methylglutaryl-CoA reductase in *Populus trichocarpa*: Overexpression of PtHMGR enhances terpenoids in transgenic poplar. Frontiers of Plant Science.

[b24-tlsr-33-2-1] Wright LP, Phillips MA, Rodríguez-Concepción M (2014). Measuring the activity of 1-deoxy-D-xylulose 5-phosphate synthase, the first enzyme in the MEP pathway, in plant extracts. Plant Isoprenoids Methods in Molecular Biology (Methods and Protocols).

[b25-tlsr-33-2-1] Yuwono SS, Faustina DR (2019). Effect of withering time and chopping size on properties of pucuk merah (Syzygium oleana) herbal tea. IOP Conference Series: Earth and Environmental Science.

[b26-tlsr-33-2-1] Zhou YJ, Gao W, Rong Q, Jin G, Chu H, Liu W, Yang W, Zhu Z, Li G, Zhu G, Huang L, Zhao ZK (2012). Modular pathway engineering of diterpenoid synthases and the mevalonic acid pathway for miltiradiene production. Journal of Americal Chemical Society.

